# Comparative genomics analysis of triatomines reveals common first line and inducible immunity-related genes and the absence of Imd canonical components among hemimetabolous arthropods

**DOI:** 10.1186/s13071-017-2561-2

**Published:** 2018-01-22

**Authors:** Federico Alonso Zumaya-Estrada, Jesús Martínez-Barnetche, Andrés Lavore, Rolando Rivera-Pomar, Mario Henry Rodríguez

**Affiliations:** 1 Centro de Investigación Sobre Enfermedades Infecciosas (CISEI), Instituto Nacional de Salud Pública, Cuernavaca, México; 2 Centro de Bioinvestigaciones (CeBio) and Centro de Investigación y Transferencia del Noroeste de Buenos Aires (CITNOBA-CONICET), Universidad Nacional del Noroeste de la Provincia de Buenos Aires, Pergamino, Argentina; 3 Laboratorio de Genética y Genómica Funcional. Centro Regional de Estudios Genómicos. Facultad de Ciencias Exactas, Universidad Nacional de La Plata, La Plata, Argentina

**Keywords:** Innate immunity, Hemipterans, Triatomines, Holometabolous, Hemimetabolous, Imd pathway

## Abstract

**Background:**

Insects operate complex humoral and cellular immune strategies to fend against invading microorganisms. The majority of these have been characterized in *Drosophila* and other dipterans. Information on hemipterans, including Triatominae vectors of Chagas disease remains incomplete and fractionated.

**Results:**

We identified putative immune-related homologs of three Triatominae vectors of Chagas disease*, Triatoma pallidipennis*, *T. dimidiata* and *T. infestans* (TTTs), using comparative transcriptomics based on established immune response gene references, in conjunction with the predicted proteomes of *Rhodnius prolixus*, *Cimex lecticularis* and *Acyrthosiphon pisum* hemimetabolous. We present a compressive description of the humoral and cellular innate immune components of these TTTs and extend the immune information of other related hemipterans. Key homologs of the constitutive and induced immunity genes were identified in all the studied hemipterans.

**Conclusions:**

Our results in the TTTs extend previous observations in other hemipterans lacking several components of the Imd signaling pathway. Comparison with other hexapods, using published data, revealed that the absence of various Imd canonical components is common in several hemimetabolous species.

**Electronic supplementary material:**

The online version of this article (10.1186/s13071-017-2561-2) contains supplementary material, which is available to authorized users.

## Background

Arthropods possess complex innate immune mechanisms to fend against viruses, bacteria, fungi and parasites. When invading microorganisms breach the cuticle and epithelial barriers, they confront humoral and cellular components of the innate immune response [1]. Intruders are recognized as non-self by pattern recognition receptors (PRRs), including peptidoglycan-binding proteins (PGRPs) and Gram-negative bacteria-binding proteins (GNBPs) [1–3]. In *Drosophila*, PRRs bind to conserved pathogen-associated molecular patterns (PAMPs) [1]. These molecular interactions initiate the immune signal transduction through three main pathways, Toll, Jak-STAT and Imd. The immune signaling culminates in the translocation into the nucleus of NF-kB/Rel transcription factors, which activate humoral responses characterized by the synthesis of antimicrobial peptides (AMPs) with broad activity spectrum against bacteria, and fungi [1, 4].

In addition to AMP production, other effector mechanisms are elicited as first line of defense, which includes coagulation, melanization and the production of nitric oxide (NO) and reactive oxygen species (ROS). Clot formation involves Hemolectin and Fondue proteins, which are critical to immobilize bacteria and initiation of wound healing [1]. Melanization is triggered by injury or recognition of microbial ligands through PRRs [5, 6]. Pro-phenoloxidase (PPO) is a precursor present in the hemolymph and hemocytes, which is activated by proteolytic cascades to phenoloxidase (PO) for *de novo* synthesis of melanin [7]. NO is a highly toxic for a wide variety of pathogens. This is produced by oxidation of L-arginine to L-citrulline by the nitric oxide synthase (Nos) [8, 9]. ROS are produced by conserved nicotinamide adenine dinucleotide phosphate (NADPH) enzymes; dual oxidase (Duox) generates hydrogen peroxide (H_2_O_2_) and hypochlorous acid, and a member of the NADPH oxidase family (Nox) generates H_2_O_2_ [10–12]. In this context, antioxidant enzymes such as catalases, glutathione peroxidases (GPx) and thioredoxin peroxidases (TPx), play important roles in cellular homeostasis [13, 14].

Alongside humoral responses, cellular responses are mediated by hemocytes [15, 16]. The main defense against viruses is RNA interference (RNAi) [17]. RNAi is based on Dicer (Dcr) and Argonaut (Ago) proteins. These mediate the production of short RNAs from double-stranded RNA (dsRNA) to guide the degradation of viral RNA by the small interfering RNA (siRNA) pathway [18, 19].

Hemocytes phagocytose bacteria, encapsulate parasites and produce immune toxic compounds for pathogen lysis [15, 16]. Internalization of invaders involves members of the scavenger receptor (SR) family and the IgSF-domain protein Dscam (Down syndrome cell adhesion molecule) [20–22]. In the hemolymph, thioester-containing proteins (TEPs) act as opsonins to mark pathogens for phagocytosis [23], C-type lectins (CTLs), recognize microbial carbohydrate structures [24, 25], and extracellular serine proteases, including CLIP-domain serine proteases (CLIPs) are activated upon recognition of aberrant tissues or microbial compounds [26].

Most information of insect immune responses has been described in *Drosophila melanogaster*, the main model organism in Diptera [1, 27, 28], but knowledge of the immune responses in hemipterans including triatomine bugs is limited [29–31]. The Toll and Jak-STAT signaling components, described in *Drosophila* have also been identified in *Rhodnius prolixus* (RPRO) (Hemiptera: Reduviidae) [32, 33], *Acyrthosiphon pisum* (ACPI) (Hemiptera: Aphididae) [30] and *Cimex lecticularis* (CLEC) (Hemiptera: Cimicidae) [31]. However, Imd signaling components, highly conserved in several insect orders [34–42], appear to be absent in RPRO and ACPI [30, 33], and information on the other components of the innate immune response in Triatomine bugs remains fractionated and incomplete [29].

In this study, we used a transcriptomic analysis to describe innate immune response genes of *Triatoma infestans* (TINF), the major vector of Chagas disease in sub-Amazonian endemic regions; *T. dimidiata* (TDIM) a vector in northern South America and Central America, extending into Mexico [43]; and *T. pallidipennis* (TPAL), an important vector in Mexico [44]. An extended comparative analysis of immune genes of TPAL, TDIM, and TINF (TTTs), and other hemipterans (RPRO, CLEC and ACPI) along with those of other holometabolous and hemimetabolous arthropods revealed the lack of several components of the Imd pathway in the hemimetabolous group.

## Methods

### Insect rearing

Colonies of *T. pallidipennis* (TPAL) (colony 0230 from Mexico), *T. dimidiata* (TDIM) (colony 0252 from Tegucigalpa, Honduras) and *T. infestans* (TINF) (colony X32 from Santiago del Estero, Argentina) established in the Centro Nacional de Chagas, Córdoba, Argentina were reared in the Centro Regional de Estudios Genómicos (CREG), Universidad Nacional de La Plata (UNLP) and the Centro de Bioinvestigaciones, Universidad Nacional del Noroeste de Buenos Aires (UNNOBA). Insects were reared at 28 °C and a partial humidity of 70% with a 12 h light/dark schedule. Insects were regularly fed using artificial feeders and chicken blood. Insect handling was performed in accordance to the World Health Organization protocol [45].

### Transcriptome preparation and sequencing

To maximize the coverage of the gene content for each species, total RNA was isolated from embryos (55) and diverse organs (reproductive and digestive tract, Malpighian tubules, brain, fat body and salivary glands) of fed and starved insects of the five nymphal stages (N1 = 8; N2 = 8; N3 = 8; N4 = 4; N5 = 4), adult mated females (4) and adult males (4) of TPAL, TDIM and TINF using Trizol (Life Technologies, Massachusetts, USA). A pool was made with 2 μg of each total RNA extraction (embryos + insect organs).

A single cDNA library for each species was independently constructed using 1.5 μg of each RNA pool (embryos + insect tissues) using the Mint-2 Kit (Evrogen, Moscow, Russia) according to the manufacturer instructions. To reduce redundancy due to highly expressed transcripts and to increase the representation of poorly represented transcripts, each library was normalized using the Trimer-2 Normalization Kit (Evrogen, Moscow, Russia) according to the manufacturer instructions. The cDNA libraries were barcoded and subjected to the shotgun sequencing protocol using the GS FLX+ (454-Roche, Connecticut, USA). Raw sequence datasets are available at the Sequence Read Archive (SRA) - NCBI: TPAL (SRX2600752), TDIM (SRX2600753) and TINF (SRX2600754).

### Data filtering, trimming and assembly

Raw reads from each barcoded library were analyzed with PRINSEQ [46] and filtered according to length, sequence complexity and quality. Each library was subjected to *de novo* assembly with the GS DeNovo assembler v.2.8 software in cDNA mode using the default parameters, and including the adaptor sequences for trimming. The assembled sequences dataset are available at the NCBI-TSA (GFMK00000000, GFMC00000000 and GFMJ01000000). The non-assembled reads were mapped to the RPRO genome (Rhodnius-prolixus-CDC_SCAFFOLDS_RproC3.fa) and proteome (Rhodnius-prolixus-CDC_PEPTIDES_RproC3.2.fa) using BLAST (Basic Local Alignment Search Tool) [47] algorithms (BLASTn and BLASTx, respectively). Non-redundant mapped reads to either database were included as singletons into the assembled dataset (full_dataset). A non-redundant database (nr_dataset) was built discarding alternative isotigs belonging to the same isogroup or unigene, by keeping the largest isotig (transcript) per isogroup. The dataset used in this work is available at http://201.131.57.23:8080/data/triatoma.

### Transcriptome completeness analysis

The assembled dataset for each species was used to identify the proportion of the core eukaryotic genome coverage. We used HMM profiles for 458 core eukaryotic proteins as provided by the Core Eukaryotic Genome Dataset (CEGMA) [48] and HMMER searches with the *hmmscan* command and the -T 40 and --domT 40 filters, as described in [49]. Following the same approach, a Benchmarking Universal Single-Copy Orthologs (BUSCO) sets for arthropod [50] was used to assess transcriptome datasets completeness.

### Comparative genomics datasets

We included three hemipteran species whole-genomes for comparison: the complete predicted-peptide sets of RPRO (Rhodnius-prolixus-CDC_PEPTIDES_RproC3.1.fa) and CLEC (Cimex-lectularius-Harlan_PEPTIDES_ClecH1.2.fa) available in VectorBase [51] and the predicted-peptide set of ACPI (aphidbase_2.1b_pep.fasta) from AphidBase [52].

A whole set of known immune-related gene sequences (“immunity-genes reference dataset”) of *D. melanogaster*, *Aedes aegypti*, *Anopheles gambiae* and other insects, including triatomines were retrieved from ImmunoDB [36], IIID [53], GenBank [54], VectorBase [51], UniProt [55], FlyBase [56] and Ensembl [57] databases. In addition, high-confidence immune-related orthologs of *Bombyx mori*, *Tribolium castaneum* and *Apis mellifera* were retrieved from published literature [35, 38, 40] (Additional file 1: Table S1).

### Immune-related homologs search

The “immunity-genes reference dataset” was used as queries to perform BLAST searches against TTTs transcriptomes and RPRO, CLEC and ACPI predicted-peptide sets. For this purpose, we used multiple BLAST algorithms (tBLASTn, BLASTn, BLASTp) using a cut-off e-value of 1.0e^−5^. BLAST outputs were retrieved, listed and compiled in the order of descending sequence identity percentage and score, and ascending e-value. Additionally, BLAST-hits with considerably short alignment lengths compared with the genes of the “immunity-genes reference dataset” were filtered. Then, the best 10 BLAST-hits were selected for detection of conserved protein-domain structures.

To search for immune-related genes of the “immunity-genes reference dataset” that produced no hits through BLAST inquiries, we conducted a tBLASTn search against all contigs and unassembled reads of the TTTs transcriptomes. Further, we performed HMM profile-based searches for those unidentified immune-related genes in TTTs and the other hemipterans (RPRO, CLEC and ACPI). We generated amino acid alignments of the unidentified immune-related genes with MUSCLE [58]. Hidden Markov models of these alignments were built using HMMER [59]. These HMM profiles were used to perform searches (*hmmscan*) against the six-frame translated sequences of the TTTs transcriptomes and the hemipteran predicted-peptide sets.

To detect conserved protein-domain structures, the immune-related sequences of TTTs and other hemipterans identified were analyzed using InterProScan [60]. The domain signatures recognized were visually inspected and compared against the genes of the “immunity-genes reference dataset” to corroborate their architecture similarities.

The immune-related homologs identified were categorized into the following major immune categories: microbial recognition and activation (GNBPs, PGRPs, CTLs, TEPs, SRs and CLIPs), signaling (Toll, Jak-STAT and Imd signaling pathways), effectors (AMPs, melanization, NO and ROS), regulation (Toll, Jak-STAT and Imd signaling regulators), antioxidant system (catalases and peroxidases), RNA interference (RNA interference machinery) and coagulation (Additional file 1: Tables S2-S8).

### Phylogenetic analysis

The amino acid sequences of defensin and lysozyme homologs identified were aligned separately using MUSCLE [58]. To compare the obtained results with previously described triatomine defensins, the sequences of defensin A (GenBank: AY196130), defensin B (GenBank: AY196131) and defensin C (GenBank: AY196132) of RPRO [61], *T. brasiliensis* (TBRA) defensin 1 (GenBank: AAV48636), defensin 2 (GenBank: ABA10770), defensin 3 (GenBank: ACH57150), defensin 4 (GenBank: ACH57151) [62, 63], and defensin (GenBank: ABD61004) of TINF were included in the alignment. Two outgroup sequences were added to the defensin dataset: defensin 1 of *A. gambiae* (VectorBase ID: AGAP01129) and defensin C of *A. aegypti* (VectorBase ID: AAEL003832). In the same way, the sequences of lysozyme A (GenBank: ABX11553), lysozyme B (GenBank: ABX11554) of RPRO [64], TBRA lysozyme 1 (GenBank: AAU04569) [62], and lysozyme 1 (GenBank: AAP83129) and lysozyme 2 (GenBank: ABI94387) [65, 66] of TINF were included in the alignment. Two outgroup sequences were added to the lysozyme dataset: lysozyme 2 of *A. gambiae* (VectorBase ID: AGAP007343) and lysozyme A of *A. aegypti* (VectorBase ID: AAEL003723). Maximum likelihood analyses were carried out using PhyML v3.0 [67] running 1000 bootstrap samples. The output trees were visualized and optimized with FigTree v1.4.3 [68].

## Results

### Transcriptome datasets metrics

Between 112 and 202 Mbp of filtered raw sequence data were generated for each triatomine species. *De novo* assembly yielded 31,175; 29,024; and 35,629 transcripts for TPAL, TDIM and TINF, respectively. Selection of the largest transcript for each isogroup (unigene) yielded non-redundant datasets with 29,789; 27,652; and 34,646 transcripts for TPAL, TDIM and TINF, respectively. BLASTx matches were identified for 71–74% of RPRO predicted proteome. Transcriptome completeness assessment for the TTTs transcriptomes indicated coverages of 86.9% in TPAL, 85.8% in TDIM and 82.2% in TINF with BUSCO [50], and higher than 90% for CEGMA dataset [48]. The datasets supporting the results of this article are available at http://201.131.57.23:8080/data/triatoma.

### Microbial recognition

#### Peptidoglycan and gram-negative bacteria-binding proteins

**Fig. 1:**
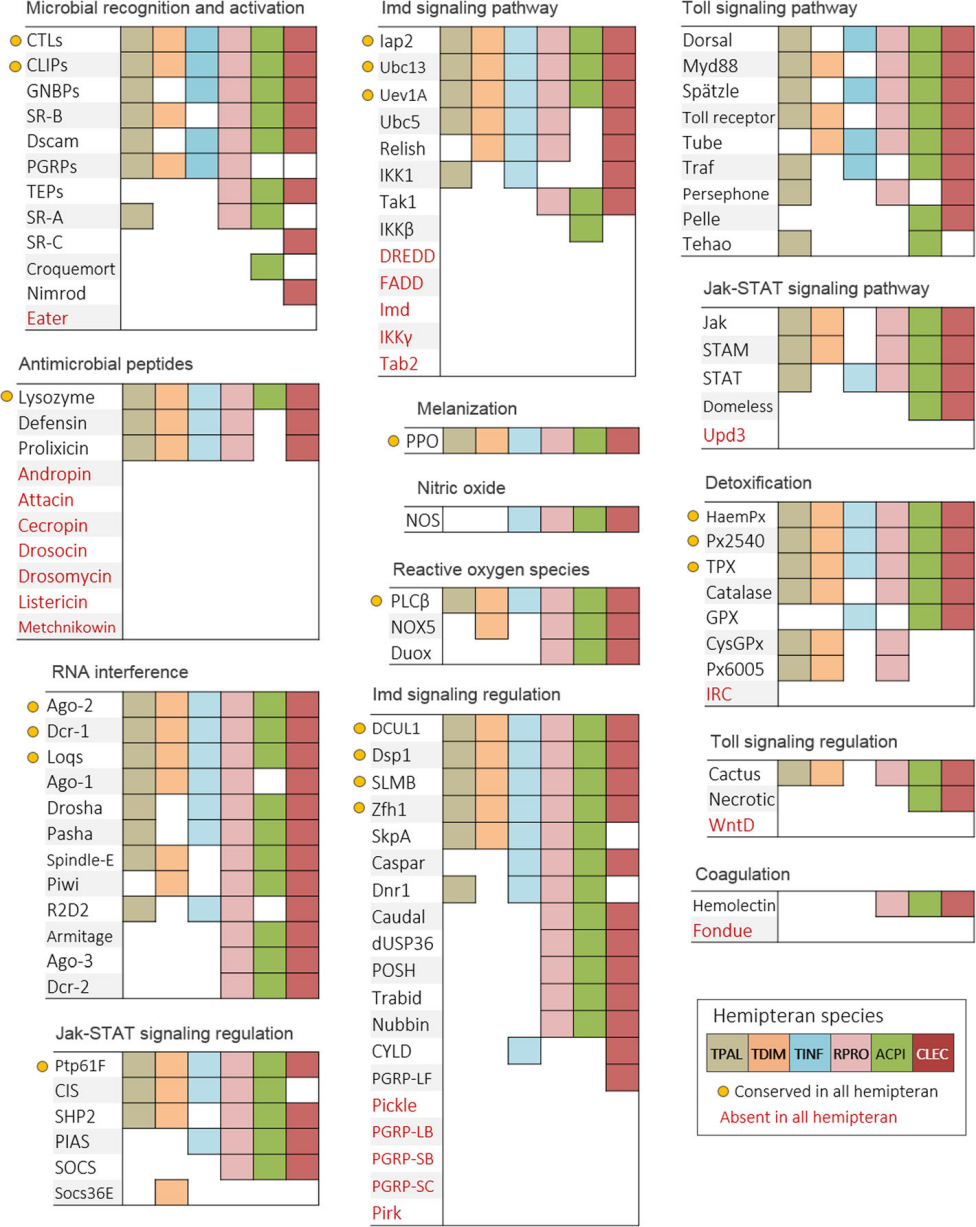
Presence/absence of immune homologs in hemipterans. Homolog tables presented according to the immune categories in which they participate. Homologs are presented in each first column (corresponding names could be seen in the abbreviations list). The presence of homologs in each species is represented by colored squares in subsequent columns. Brown squares represent *T. pallidipennis* (TPAL), orange *T. dimidiata* (TDIM), blue *T. infestans* (TINF), pink *R. prolixus* (RPRO), green *A. pisum* (ACPI) and red *C. lecticularis* (CLEC). The absence of homologs corresponds to blank squares/spaces. The conserved immune-related homologs in all the hemipterans are indicated with yellow circles. Names in red font correspond to immune-related homologs absent in all hemipterans

We identified PGRP-like homologs containing both peptidoglycan recognition protein (IPR015510) and N-acetylmuramoyl-L-alanine amidase (IPR002502) domains in TTTs, and the presence of three PGRP with identical protein signatures in RPRO, which were previously identified [33] (Fig. 1, Additional file 1: Table S2). We also recognized a PGRP-like gene (CLEC005283) in CLEC (PGRP-LF), which was previously related to functions other than microbial recognition [31]. As previously reported, we were unable to detect PGRP-like genes in ACPI [30].

GNBP-like homologs with beta-1,3-glucan-binding N-terminal (IPR031756) and/or concanavalin A-like lectin/glucanase (IPR013320) domains were observed in all hemipterans, except TDIM (Fig. 1, Additional file 1: Table S2).

#### Lectins

Several CTLs homologs containing C-type lectin fold (IPR016187) domains were identified in all hemipterans (Fig. 1, Additional file 1: Table S2).

#### Thioester-containing proteins

Notably, we could not identify TEPs in TTTs; however, one TEP encoding gene containing immunoglobulin E-set (IPR001599) and α2-macroglobulin thiol-ester bond-forming (IPR019565) domains were detected in RPRO and at least two different TEP homologs with similar protein signatures were observed in CLEC and ACPI (Fig. 1, Additional file 1: Table S2).

#### Cellular receptors

We detected homologs containing a *Dscam*-domain (IPR033027) in RPRO, CLEC and ACPI. Conversely, sequences lacking this domain but containing an immunoglobulin-like fold (IPR013783) and a fibronectin type III (IPR003961) domains were identified in TPAL and TINF.

Scavenger receptors class B (SR-B) homologs, characterized by a CD36 (IPR002159) domain were the most abundant SR class in all hemipterans, except TINF. The SR-B croquemort receptor was not detected in any species, except ACPI (Fig. 1, Additional file 1: Table S2). Scavenger receptors class C (SR-C) encoding genes were identified exclusively in CLEC. However, only one of them (CLEC000453) contains the characteristic extracellular sushi/SCR/CCP (IPR000436) domain (Fig. 1, Additional file 1: Table S2).

#### CLIP-serine proteases

We identified sequences containing serine protease (IPR001254) and peptidase S1 (IPR009003) domains in all hemipterans. However, homolog genes with a proteinase regulatory CLIP (IPR022700) domain were observed only in RPRO (RPRC003090; RPRC009383) and CLEC (CLEC010998; CLEC006847; CLEC001617) (Fig. 1, Additional file 1: Table S2).

### Signaling

#### Toll signaling pathway

**Fig. 2:**
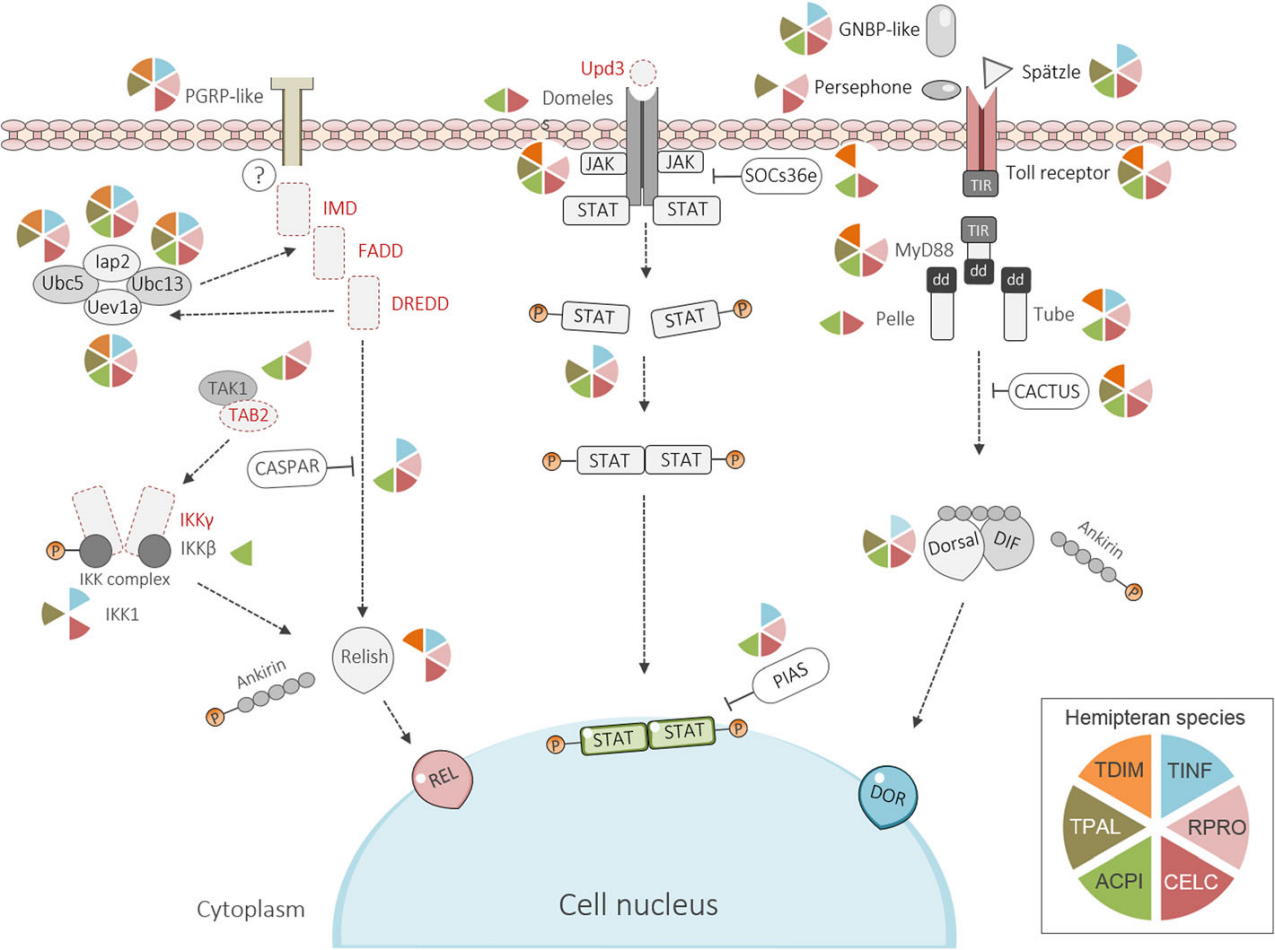
Immune signaling pathways. Diagram of the Imd, Jak-STAT and Toll signaling pathways according to their homolog members identified in the hemipterans. The color-divided pies indicate the presence of the immune signaling homologs among the six species. Brown represents *T. pallidipennis* (TPAL), orange *T. dimidiata* (TDIM), blue *T. infestans* (TINF), pink *R. prolixus* (RPRO), green *A. pisum* (ACPI) and red *C. lecticularis* (CLEC). Names in red font correspond to immune-related homologs absent in all hemipterans

Previously, several Toll signaling proteins were reported in RPRO [32, 33] and we recognized most of the canonical components of the Toll signaling pathway in triatomine bugs (TTTs and RPRO). Of these, we detected homologs of the extracellular cytokine spätzle, the Toll receptor, the death-domain containing adaptor proteins Myd88 and Tube, and the NF-kB/Rel transcription factor Dorsal (Fig. 2, Additional file 1: Table S3). We also corroborated that canonical components of this signal cascade are conserved in CLEC and ACPI [30, 31] (Fig. 2, Additional file 1: Table S3). In addition, we identified homologs of Cactus in most hemipterans; necrotic homologs were identified only in CLEC and ACPI but wntD was not found in any hemipteran. These molecules are responsible for the negative regulation of the Toll pathway in the absence of pathogenic challenges (Fig. 1, Additional file 1: Table S5).

#### Jak-STAT signaling pathway

Domeless receptor homologs with both fibronectin type III and immunoglobulin-like fold domains were detected only in CLEC and ACPI (Fig. 2, Additional file 1: Table S3). We did not detect homologs of the hemocyte-released cytokine Unpaired-3 (Upd-3) in any of the hemipterans. However, homologs of Janus kinase (Jak), containing a serine-threonine/tyrosine-protein kinase catalytic domain (IPR001245) and homologs of STAT with transcription factor STAT domain (IPR001217) were observed in all hemipterans, except TINF and TDIM, respectively.

We also identified several homologs of negative regulators, as SOCS36E, PIAS and Ptp61F. Of these, homologs of Ptp61F with a PTPase (IPR000242) domain were present in all hemipterans (Fig. 1, Additional file 1: Table S5).

#### Imd signaling pathway

We were unable to detect key components of the Imd pathway in all hemipteran species. Of these, as described for ACPI, RPRO and CLEC, the death-domain protein Imd, FADD (Fas-Associated protein with death-domain) and the caspase DREDD (death-related ced-3/Nedd2-like protein) were not detected in any TTT species. However, homologs the three E2 ubiquitin conjugating enzymes: Uev1a, Ubc13 (Bendless), Ubc5 (Effete) and the E3 ubiquitin ligase Inhibitor of Apoptosis Protein (IAP2) were present in all hemipterans, except for Effete in ACPI (Fig. 2, Additional file 1: Table S3). Homologs of the NF-kB/Rel transcription factor Relish, with Rel homology, DNA-binding domain (IPR011539) were observed in CLEC and all triatomine bugs, except TPAL. As previously reported, we were unable to detect Relish genes in ACPI [30] (Fig. 2, Additional file 1: Table S3).

We detected several negative regulators of the Imd signaling cascade. Homologs of Caspar with UBX (IPR001012) and UAS (IPR006577) domains were identified in all hemipterans, except TPAL and TDIM (Fig. 1, Additional file 1: Table S5). Homologs that alter the stability of the Imd pathway members such as dUSP36 and POSH were observed in ACPI, CLEC and RPRO, but not in TTTs. In addition, all components of the SKPA/SLMB/DSL1 complex were identified in all hemipterans, except for SKPA in CLEC (Fig. 1, Additional file 1: Table S5).

Homologs of the transcriptional repressor Zfh1 (Zn finger homeodomain 1) with zinc finger C2H2-type (IPR013087) and homeobox (IPR001356) domains were identified in all hemipterans. Similarly, homologs of the caspase inhibitor Dnr1 (Defense repressor 1) were not identified only in TDIM and CLEC (Fig. 1, Additional file 1: Table S5). While homologs of the transcription factor caudal with a helix-turn-helix (IPR000047) motif and multiple homeobox (IPR001356; IPR020479; IPR009057; IPR017970) domains were observed in all hemipterans, but not in TTTs (Fig. 1, Additional file 1: Table S5). Likewise, homologs of the deubiquitinase Trabid [69] with zinc finger, RanBP2-type (IPR001876) and OTU (IPR003323) domains were present in RPRO and ACPI. A Trabid homolog sequence containing an OTU domain, but not zinc finger motifs, was detected in CLEC. No Trabid homologs were detected in TTTs. While homologs of Pickle, a *Drosophila* negative regulator that selectively inhibit Relish [70] were not detected in any hemipteran (Fig. 1, Additional file 1: Table S5).

### Immune effectors

#### Antimicrobial peptides

**Fig. 3:**
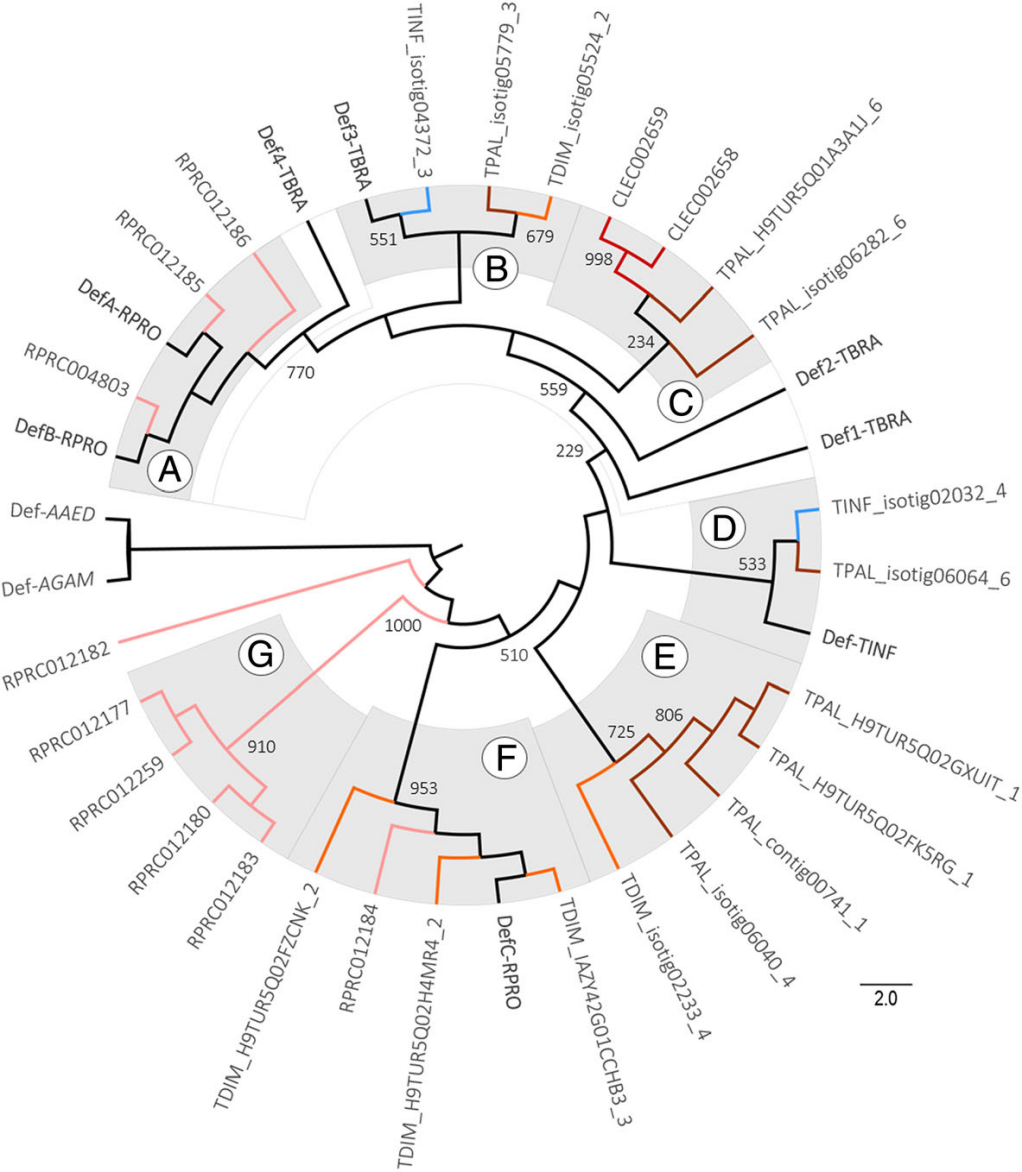
Defensin phylogenetic analysis. Maximum Likelihood cladogram of hemipteran defensins. Branch color indicates the different hemipteran species. Brown branches correspond to *T. pallidipennis* (TPAL), orange to *T. dimidiata* (TDIM), blue to *T. infestans* (TINF), pink to *R. prolixus* (RPRO) and red to *C. lecticularis* (CLEC). No defensin homologs were identified in *A. pisum* (ACPI). The black color branches correspond to genes encoding defensin A, B and C of RPRO (DefA-C-RPRO), defensin 1, 2, 3 and 4 of *T. brasiliensis* (TBRA) (Def1-4-TBRA) and defensin of TINF (Def-TINF) as well as defensin sequences of *A. gambiae* (Def-AGAM) and *A. aegypti* (Def-AAED) that were used as outgroups. The gray shading enclosing the branches represents the clusters formed. Branch labels show the bootstrap samples supporting that branch

A number of defensin homologs, containing both knottin scorpion toxin-like (IPR003614) and defensin invertebrate/fungal (IPR001542) domains, were identified in all hemipterans, except ACPI (Fig. 1, Additional file 1: Table S4). ACPI genome apparently has no genes encoding for defensins [30]. The maximum likelihood tree, based on defensin homolog sequences, showed the presence of seven clades (A-G) (Fig. 3). In clade A, genes encoding defensin A and B of RPRO (DefA-RPRO, DefB-RPRO) were grouped with three sequences of RPRO (RPRC004803, RPRC012185 and RPRC012186) with high bootstrap support (770). A gene encoding defensin 4 of TBRA (Def4-TBRA) was located at the basal position of this clade. In clade B, a gene encoding defensin 3 of TBRA (Def3-TBRA) was grouped with defensin sequences of TINF (TINF_isotig04372_3), TPAL (TPAL_isotig05779_3) and TDIM (TDIM_isotig05524_2) with medium bootstrap support values (551–679). In clade C, both defensin homolog sequences of CLEC (CLEC002659 and CLEC002658) were grouped with two TPAL sequences (TPAL_H9TUR5Q01A3A1J_6 and TPAL_isotig06282_6) with variable bootstrap values (234–998). Two genes encoding defensins of TBRA (Def1-TBRA and Def2-TBRA) were located at the basal position of the A, B and C clades, with variable bootstrap values (229–559). In clade D, a defensin from the gut of TINF (Def-TINF) was grouped with a TINF (TINF_isotig02032_4) and TPAL (TPAL_isotig06064_6) sequences with medium bootstrap support (533). Clade E grouped four TPAL sequences (TPAL_H9TUR5Q02GXUIT_1, TPAL_H9TUR5Q02FK5RG_1, TPAL_contig00741_1, and TPAL_isotig06040_4) and a sequence of TDIM (TDIM_isotig02233_4) with a bootstrap support value of 725. Clade F grouped a gene encoding defensin C of RPRO (DefC-RPRO) with another sequence of RPRO (RPRC012184), as well as three TDIM sequences (TDIM_IAZY42G01CCHB3_3, TDIM_H9TUR5Q02H4MR4_2 and TDIM_H9TUR5Q02FZCNK_2) with high bootstrap support (953). In clade G, four sequences of RPRO (RPRC012183, RPRC012180, RPRC012259 and RPRC012177) grouped separately from other defensin sequences of RPRO and were supported by high bootstrap value (1000). A RPRO sequence (RPRC012182) was located separate from the other defensin clades. Dipteran defensin 1 of *A. gambiae* (Def-AGAM) and defensin C of *A. aegypti* (Def-AAED) were clustered in an outgroup branch.

**Fig. 4:**
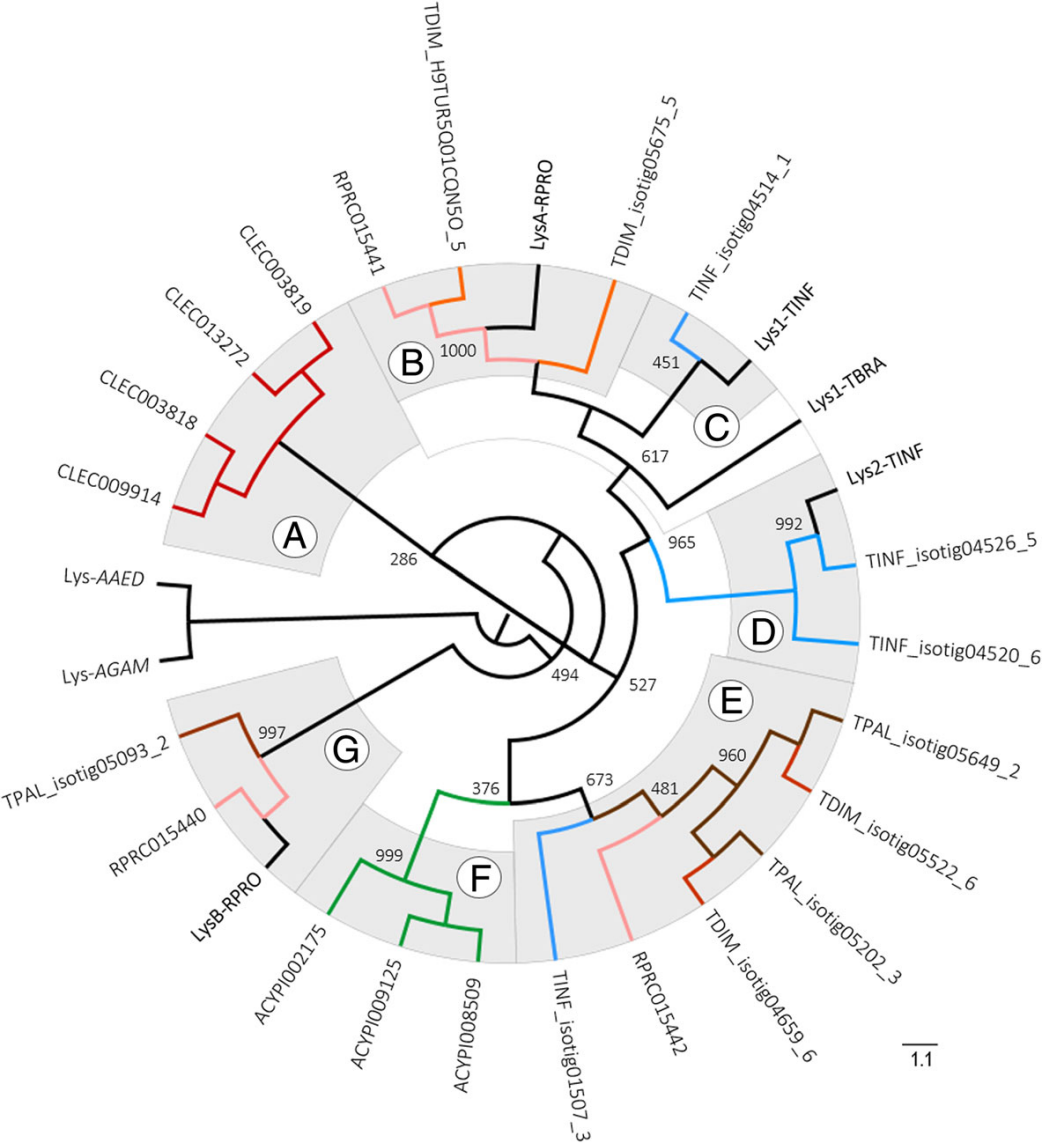
Lysozyme phylogenetic analysis. Maximum Likelihood cladogram of hemipteran lysozymes. Branch colors indicate the different hemipteran species. Brown branches correspond to *T. pallidipennis* (TPAL), orange to *T. dimidiata* (TDIM), blue to *T. infestans* (TINF), pink to *R. prolixus* (RPRO), red to *C. lecticularis* (CLEC) and green to *A. pisum* (ACPI). The black branches correspond to genes encoding lysozyme A and B of RPRO (LysA, B-RPRO), lysozyme 1 of *T. brasiliensis* (TBRA) (Lys1-TBRA) and lysozyme 1 and 2 of TINF (Lys1-TINF) as well as lysozyme sequences of *A. gambiae* (Lys-AGAM) and *A. aegypti* (Lys-AAED), which were used as outgroups. The gray shading enclosing the branches represents the clusters formed. Branch labels show the bootstrap samples supporting that branch

Lysozyme homologs, containing Lysozyme-like (IPR023346) domains, were identified in all hemipterans (Fig. 1, Additional file 1: Table S4). The maximum likelihood tree, based on lysozyme homolog sequences, showed the presence of seven clades (A-G) (Fig. 4). In clade A, all lysozyme homolog sequences of CLEC (CLEC009914, CLEC003818, CLEC013272 and CLEC003819) were grouped with low bootstrap support (286). Clade B, grouped a gene encoding lysozyme A of RPRO (LysA-RPRO) with one sequence each of RPRO (RPRC015441) and TDIM (TDIM_H9TUR5Q01CQN5O_5) with high bootstrap support (1000). A sequence of TDIM (TDIM_isotig05675_5) was located at the basal position of this clade. In clade C, a gene encoding lysozyme 1 of TINF (Lys1-TINF) and a sequence of TINF (TINF_isotig04514_1) were grouped with medium bootstrap support (451). A gene encoding lysozyme 1 of TBRA (Lys1-TBRA) was located at the basal position of the B and C clades, with medium bootstrap support value (617). In clade D, a gene encoding lysozyme 2 of TINF (Lys2-TINF) was grouped with two sequences of TINF (TINF_isotig04526_5 and TINF_isotig04520_6), with high bootstrap support values (965–992). Clade E clustered sequences of TPAL (TPAL_isotig05649_2 and TPAL_isotig05202_3) and TDIM (TDIM_isotig05522_6, TDIM_isotig04659_6) with high bootstrap support (960). A RPRO (RPRC015442) and TINF (TINF_isotig01507_3) sequences were located at the basal position of this clade, with medium bootstrap support (481–673). All lysozyme homolog sequences of ACPI (ACYPI008509, ACYPI009125 and ACYPI002175) were grouped with variable bootstrap values (376–999) in clade F. Clade G clustered a gene encoding lysozyme B of RPRO (LysB-RPRO) and RPRO (RPRC015440) and TPAL (TPAL_isotig05093_2) sequences, with variable bootstrap support (494–997). Dipteran lysozyme 2 of *A. gambiae* (Lys-AGAM) and lysozyme A of *A. aegypti* (Lys-AAED) were clustered in an outgroup branch.

We also identified prolixicin homologs in all triatomine bugs and CLEC, but we failed to detect prolixicin homologs in ACPI (Fig. 1, Additional file 1: Table S4).

No homologs of cecropin, attacin, andropin, drosomycin, drosocin, listericin and metchnikowin were identified in any hemipteran (Fig. 1, Additional file 1: Table S4).

#### Melanization

We found PPO homolog sequences, containing hemocyanin C-terminal (IPR005203) and immunoglobulin E-set (IPR014756) domains, in TTTs (Fig. 1, Additional file 1: Table S4). We corroborate the presence of previously identified PPO homologs, containing the aforementioned domains and a tyrosinase copper-binding (IPR002227) domain, in RPRO, CLEC and ACPI [30, 31, 33] (Fig. 1, Additional file 1: Table S4).

#### Nitric oxide

We identified a nitric oxide synthase (Nos) homolog sequence, containing a nitric oxide synthase N-terminal (IPR004030) domain, in TINF, but not in TDIM and TPAL. Also, Nos homologs with a eukaryote nitric-oxide synthase (IPR012144) domain, were identified in RPRO, CLEC and ACPI (Fig. 1, Additional file 1: Table S4).

#### Reactive oxygen species

Duox homologs were not detected in TTTs. But, we identified homolog sequences, containing a Dual oxidase peroxidase (IPR034821) and Haem peroxidase (IPR010255; IPR019791) domains, in RPRO and ACPI; as well as a number of previously reported homologs of Duox enzymes in CLEC [31]; although, only one (CLEC009522) of these sequences contains a Haem peroxidase and a Dual oxidase peroxidase domains (Fig. 1, Additional file 1: Table S4).

Homologs of NADPH oxidase family (Nox), containing (NADPH) oxidase 5 (IPR029648) domains, were identified in all hemipterans except TPAL and TINF (Additional file 1: Table S4). In addition, we detected homologs of the phospholipase C-β (PLCβ), containing phosphoinositide phospholipase C (IPR001192) domains, in all hemipterans (Fig. 1, Additional file 1: Table S4).

### Antioxidant system

#### Antioxidant system

Homologs of a *Drosophila* catalase, with multiple catalase-like and catalase immune-responsive (IPR010582) domains were observed in all hemipterans, except TINF (Fig. 1, Additional file 1: Table S6). CysGPx homologs (that participate in the elimination of hydrogen and organic peroxides), containing both glutathione peroxidase (IPR000889) and thioredoxin-like fold (IPR012336) domains, were detected only in TPAL and TDIM. Several TPx homologs, containing thioredoxin-like fold and peroxiredoxin C-terminal (IPR019479) domains, were identified in all hemipterans. Additionally, numerous Haem peroxidase homologs, with peroxidase (IPR010255; IPR019791) domains and *Drosophila* peroxiredoxin-2540 homolog sequences, containing both alkyl hydroperoxide reductase subunit C/thiol specific antioxidant (IPR000866) and peroxiredoxin C-terminal (IPR019479) domains, were detected in all hemipterans (Fig. 1, Additional file 1: Table S6).

### RNA interference

#### siRNA pathway

We recognized key components of the siRNA pathway in all hemipterans. Of these, Dcr-2 homologs, with Dicer dimerization (IPR005034), helicase ATP-binding (IPR014001), P-loop NTPase (IPR027417), PAZ (IPR003100) and ribonuclease III (IPR000999) domains, were identified in RPRO, CLEC and ACPI, but not in TTTs. R2D2 homologs, with a double-stranded RNA-binding (IPR014720) domains, were observed in CLEC and all triatomine bugs except TDIM. In triatomine bugs, we identified Ago2 homologs with Piwi (IPR003165), ribonuclease H-like (IPR012337) and PAZ domains. While homologs of these molecules containing other Ago-related (IPR032474; IPR014811; IPR032472) domains were detected in CLEC and ACPI (Fig. 1, Additional file 1: Table S7).

We also identified double-stranded RNA-binding domain Loqs homologs in TPAL, TDIM, and ACPI, while sequences with the characteristic staufen C-terminal (IPR032478) domain were observed in TINF, RPRO and CLEC. Additionally, other key enzymes from several RNAi-related pathways were detected in all hemipterans (Fig. 1, Additional file 1: Table S7).

### Coagulation

We detected *Hemolectin* homologs, containing both coagulation factor 5/8 C-terminal (IPR000421) and von Willebrand factor, type D (IPR001846) domains, in RPRO, CLEC and ACPI, but not in TTTs. Homolog sequences of Fondue were not identified in any hemipteran (Fig. 1, Additional file 1: Table S8).

## Discussion

Here, using as reference immune molecules described in established invertebrate immunology models [1, 36], we present a compressive description of the humoral and cellular innate immune components of four important Chagas disease vectors (TPAL, TDIM, TINF and RPRO), along with two other hemipterans phylogenetically related (CLEC and ACPI). Key homologs of constitutive and induced immune responses were identified in all the studied hemipterans (Fig. 1). However, compared to other insects; important differences were observed (Fig. 2). Our results in the TTTs extend previous observations in other hemipterans lacking several components of the Imd signaling pathway. Further comparison with other hexapods, using published data, revealed that lacking Imd canonical components is common in several hemimetabolous species.

Transcriptome analysis of organisms with no sequenced genomes could provide useful preliminary gene catalogues. Although we cannot exclude that genes expressed at low levels or restricted to few cells could not be detected, this possibility was reduced by the normalization of the libraries and their high coverage values assessed using two approaches (CEGMA and BUSCO). Almost two-thirds of the *de novo* TTT transcriptomes mapped to the RPRO genome and predicted proteome [33], and three quarters of the RPRO proteome had homologous matches in TTT datasets. Nevertheless, although the constructed TTT-immune landscape appears patchy, the presence of canonical immune homologs in at least one of the triatomines could be considered as extant in TTTs as a group; this was further supported by their presence in the RPRO genome. While their presence only in RPRO (different genera) could not be interpreted as present in all triatomine bugs. The use of the available genomes of CLEC [31] and ACPI [71] ensured the most complete data available for meaningful comparative analysis with other hemipterans.

Overall, we identified hemipteran homologs belonging to the major immune categories (microbial recognition and activation, signaling, effectors, regulation, antioxidant system, RNA interference and coagulation), but with particular compositions that could be attributed to lifestyle and environmental exposures of these insects.

Conserved microbial recognition GNBPs in all hemipterans is consistent with the long ancestry of these receptors, while the presence of PGRPs in all hematophagous hemipterans, but not in ACPI could be indicative of their involvement in surveillance and activation of immune signaling against pathogens encountered in their different environs. Likewise, hemipterans possess cellular receptors as Dscam and different classes of scavenger receptors involved in cellular internalization of foreign agents [20, 21]. This hemocyte response also involves CTLs that are conserved in hemipterans, and participate in the defense against flagellated parasites [72, 73]. In triatomine bugs, these pathogen-binding molecules facilitate the recruitment of hemocytes for the encapsulation and melanization of pathogens [74].

TEPs share similarities with vertebrate complement factors C3/C4/C5 and have thioester sites for microbial recognition in a common [23]. TEP1 of *A. gambiae* participates in the defense against bacterial and *Plasmodium* infections [75, 76]. At least one member of the TEP family was detected in RPRO, CLEC and ACPI, but none in TTTs, which reflects an apparent low proportion of these recognition-molecules in hemipterans [30, 77, 78]. In contrast, TEPs are subject to rapid lineage-expansions in other insects such as *Drosophila* [34] and mosquitoes [37, 79]. Remarkably, *Musca domestica* possesses the largest TEP repertoire of the sequenced dipterans. This genic expansion is related to the coexistence of these insects with a wide diversity of microorganisms [80]; the low representation of TEPs in hemipterans could be related to a limited exposure to pathogens mainly due to restricted diets.

Similarly, although we detected several serine proteases in all hemipterans, serine proteases with CLIP domain were detected in low proportions. In other insects, CLIPs participate in the regulation of extracellular pathways involved in the proteolytic activation of PPO and the Toll signaling pathway [81, 82], and represent large families of genes [36–38, 83]. The relative paucity of CLIP-domain contrast with an enrichment of protease inhibitor such as pacifastins (IPR008037), previously detected in *Triatoma* species (Martinez-Barnetche et al., unpublished observations). In arthropods, pacifastins are upregulated after immune challenge and act as downregulators of the melanization by preventing PO activation [84]. Nevertheless, the significance of this finding remains uncertain.

As expected, genes responsible for constitutive primary effector mechanisms, widely conserved in insects, such as PPO enzymes responsible for the *de novo* synthesis of melanin, were present in all hemipterans. Melanization plays an important role in the elimination of a variety of pathogens, as well as in facilitating wound healing [85, 86]. In addition, this reaction is related to hemocyte-mediated processes such as phagocytosis of bacteria and parasite encapsulation [87]. We also detected PLCβ homologs and numerous NADPH enzymes, responsible for the production of ROS. In triatomines, oxygen intermediates constitute a primary defense line against trypanosomatid parasites [88, 89]. ROS also play a key role in the regulation of intestinal bacteria, which undergo dramatic increases after blood meals [90]. In this context, antioxidants are particularly important for hematophagous insects continuously exposed to ROS, due to the release of heme after blood-feeding [88]. On the other hand, prolonged exposure to ROS leads to oxidative stress and cell damage [91], and the presence of enzymes responsible for the removal of hydrogen and organic peroxides, such as catalases, GPx and TPx, indicates the important role of these redox mechanisms for cellular homeostasis in hemipterans.

As in other insects, in hemipterans NO could act as a signaling and cytotoxic molecule after the damage produced by bacteria and parasites [8, 92–94]. NO is active in the hemolymph and the digestive tract of triatomines, where it contributes to resists trypanosomatid parasites [9, 94]. In addition, NO triggers the production of other effector molecules such as AMPs [8, 92, 95].

The synthesis of AMPs is consequence of the activation of NF-kB and it is the hallmark of the induced humoral immune response in insects [4, 27]. As in other insects, the role of AMPs in triatomines is the defense against microbial agents, including *T. cruzi* [64]. Except for ACPI, we found defensin homologs in most hemipterans. The phylogenetic analysis revealed divergence between defensins homologs expressed in different tissues among species (Fig. 3). A group of RPRO defensins (clade A) appears related to DefA and DefB of RPRO that are upregulated in the fat body and midgut after immune challenge [61]. Similarity, a set of defensin sequences of TDIM and RPRO (clade F) seems related to DefC of RPRO that is also involved to immune functions [61]. While, a cluster of sequences of TINF, TPAL and TDIM (clade B and C) appears related to TBRA defensins (Def1–3) which are induced in the triatomine salivary glands and digestive tract after feeding [62, 63]. Both CLEC defensins appear to be related with TPAL sequences (clade C). Other not previously described TINF and TPAL sequences (clade D) grouped with a defensin expressed in the gut of TINF. Interestingly, a RPRO basal taxon (RPRC012182) and a set of defensins of this species (clade G) were grouped apart from other hemipterans, including the rest of RPRO sequences. A similar separation pattern was observed for a group of TPAL and TDIM sequences (clade E) that seem closely related, although the role of these groups of defensins is unclear.

Prolixicin homologs were only identified in triatomine bugs and CLEC. This AMP, related to the diptericin-attacin family, is expressed by the fat body and midgut of triatomines in response to bacterial infections, although it is not toxic for *T. cruzi* [96]. Lysozymes were conserved in all hemipterans. The function of lysozyme is still not clear in triatomines. These enzymes exhibit organ-dependent expression and are reportedly involved in both digestive and immune functions [64–66]. The phylogenetic analysis exposed separate clusters of CLEC (clade A), ACPI (clade F) and TTTs lysozyme sequences that seem to be related to their digestive and immune defense functions (Fig. 4). A group of RPRO and TDIM sequences appears related to LysA of RPRO associated to immune-related functions (clade B). This molecule is predominantly expressed in the intestinal tract after ingestion of *T. cruzi* in a blood meal, and after injection of bacteria into the haemocel [63, 64]. LysB of RPRO that is expressed primarily in hemocytes and fat body after bacterial challenge [64] grouped with other not previously described RPRO and TPAL sequences (clade G) indicating their possible participation in immunity. A TINF lysozyme sequence, appears related to Lys1 of TINF and Lys1 of TBRA (clade C), which are upregulated in the stomach after feeding [62, 65]. This upregulation may reflect their digestive functions or their induction in response to the drastic increase of bacterial populations in this organ after a blood meal [90]. Other two TINF lysozyme sequences clustered with Lys2 (clade D) expressed in the midgut of TINF, with not yet elucidated its physiological function [66]. While, a set of TPAL, TDIM, TINF and RPRO lysozymes grouped separately (clade E) from the other sequences, but their roles is still unknown.

In contrast, no homologs of other *Drosophila* AMPs such as cecropin, attacin, andropin, drosomycin, drosocin, listericin and metchnikowin were identified in any hemipteran, corroborating previous observations in RPRO [97]. Although, the majority of AMPs, such as defensins, cecropins, proline-rich peptides and attacins have been found in several insect orders [98], some AMPs have been identified only in certain orders [99, 100]. While some AMPs, such as cecropins of *M. domestica,* exhibit significant duplication rates [101], it is possible that differences in insect AMPs repertoires could be consequence of different selection pressures exerted by exposure to pathogens and habitat conditions.

The Toll and Jak-STAT signaling cascades with most of their canonical components were documented in all hemipterans, corroborating previous observations in the RPRO, CLEC and ACPI genomes [30–33]. These ancestral pathways are widely conserved and participate in the development of bilaterally symmetric animals (such as worms, mollusks, arthropods and vertebrates) [102, 103]. In *Drosophila*, both Toll and Jak-STAT pathways serve a dual function in development and immunity [1].

Conversely, signaling canonical components Imd, FADD and DREED of the Imd pathway were not detectable in all studied the triatomines. The Imd signaling pathway is responsible for intestinal immune responses in dipterans. In *Drosophila*, intestinal diptericin, cecropin, drosocin and attacin regulated by this pathway, are constitutively expressed [104, 105], and the gut microbiota maintains basal their expression levels [104, 106, 107]. In mosquitoes, the growth of gut-dwelling bacteria induced by blood meals increases their expression through activation of Imd [105]. Thus, although we did not include microbial challenged insects in the preparations of our transcriptomes, blood-fed individual were included, and along with a basal expression, we expect similar inductions in these insects. As transcriptome datasets were normalized, the possibility that our transcriptomes were unable to detect at least one transcript of Imd pathway canonical members was minimal. The absence of these molecules in TTT is consistent previous observations in the RPRO, CLEC and ACPI genomes [30, 31, 33], which we corroborated by examining these genomes datasets.

Orthologs of Imd, FADD and DREED are highly conserved in a number of holometabolous insects from the orders Diptera, Hymenoptera, Lepidoptera and Coleoptera [34–42]; although these insects exhibit considerable variations in the size and diversity of immune gene families [35–37]. In contrast, the absence of these Imd components appears to be a common feature in insects with incomplete metamorphosis (hemimetabolous), such as *Anasa tristis* (Hemiptera: Coreidae) [108], *Diaphorina citri* (Hemiptera: Liviidae) [77, 109], *Bemisia tabaci* (Hemiptera: Aleyrodidae) [110, 111] and *Pediculus humanus* (Phthiraptera: Pediculidae) [112]. Even in chelicerates (*Tetranychus*, *Metaseiulus* and *Ixodes*) [113, 114] and ametabolous hexapods of the subclass Collembola (*Folsomia candida* and *Orchesella cincta*) (without morphological transformations during their development) [115] appear to lack these key immune signaling molecules.

It has been suggested that the absence of key immune signaling components, particularly in the Imd pathway, may be the result of largely free-of-microbes diets (phloem sap or blood) that do not require specific defenses within the digestive tract, and do not exert selective pressures to maintain the high cost of immune defense [30].

In addition, these insects need to harbor populations of obligate symbionts that synthesize essential amino acids and vitamins that are poorly represented in their restricted diets [30, 116–119]. In triatomines, extracellular symbionts are mainly acquired through the consumption of feces of conspecifics during their first life stages and inhabit the midgut lumen, where they play digestive roles (hemolysis) [90, 120]. As depletion of symbionts results in drastic physiological and pathological alterations [121], it has been suggested that lacking a complete Imd (mainly responsible for the intestinal immune response) is an adaptation to ensure functional symbiosis. Nevertheless, other insects with obligate symbiotic relationships employ AMPs as coleoptericin to control symbiont populations [122, 123]. The induction of the coleoptericin family members is mainly regulated by the Imd pathway [124].

Hemimetabolous insects hatch as nymphs, morphologically similar to adults and grow progressively through molts until the adult stage. Adults differ from nymphs for the presence of functional wings and genitalia. In contrast, the holometabolous insects hatch as larvae and undergo drastic anatomical changes to pupa and adult. Among these, they suffer the complete remodeling of the larval midgut, which is then replaced by a new pupal epithelium that matures to the adult epithelium [125]. The destruction of larval intestinal epithelium is directed by the hormone 20-hydroxyecdysone (ecdysone) and mediated by processes of programmed cell death, involving the activation of caspases and regulation of IAP2, a key component of the Imd pathway [126]. In *Drosophila*, Imd is expressed at high levels during the pupariation stage, when massive apoptotic events occur [127].

The overexpression of Imd results in the activation of *reaper* in adult flies [127]. *Reaper* is a key pro-apoptotic gene in *Drosophila* [128]. Induction of *reaper* occurs in a stage-specific manner during larval midgut histolysis [126]. At this time, intestinal cells are exposed to the microorganisms present in the gut during the larva-pupa transition, coinciding with the release of different antimicrobial components into the intestine [129–132]. This may also contribute to the protection of the pupa and the adult from the bacterial threats that could originate during midgut remodeling [130]. This intestinal immune process is thought to be regulated, but is still not described. Although other proteins linked to the Imd cascade were identified in hemipterans, these may represent homologs involved in more general cellular processes such as ubiquitination and apoptosis [126, 133], but are not true Imd pathway orthologs. Thus, we speculate that these proteins, along new components (Imd, FADD, DREDD) that constitute the Imd signaling pathway were recruited by holometabolous insects in response to pathogenic bacterial threats during the intestinal remodeling in the course of metamorphosis. This concept is phylogenetically sound, as ametabolous and hemimetabolous insects (lacking Imd) are more ancient than holometabolous insects [134, 135].

Although it is difficult to establish the absence of genes from transcriptomes, the completeness of our assemblies (BUSCO and CEGMA assessments), and the datasets normalization from insects expected to respond to midgut microbiota, support the absence of Imd components in TTTs. Nevertheless, although Imd, FADD and DREED are absent in the genomes of another Reduviid (RPRO) and the hemipterans CLEC and ACPI, further evidence is needed to corroborate our assumption. The high-resolution genomic-scale data derived from 1 K (Insect Transcriptome Evolution) with more than a thousand of insect-transcriptomes from all recognized taxonomic orders [135] and the 5000 arthropod genomes initiative (i5K) may represent useful resources to prove our hypothesis [136].

## Conclusions

We provide evidence for the presence of major constitutive and inducible immune components in four important Chagas disease vectors (*T. pallidipennis*, *T. dimidiata*, *T. infestans* and *R. prolixus*) and two related hemipterans (*C. lecticularis* and *A. pisum*). Homologs involved in microbial recognition and immune activation (GNBPs, PGRPs, CTLs, TEPs, SRs and CLIPs) were documented in most species. But differences, like low proportions of TEPs and CLIPs, attributable to lifestyle and limited pathogen exposure were observed.

Conserved constitutive immune components responsible for *de novo* synthesis of melanin (PO), nitric oxide (Nos), and ROS production (PLCβ and NADPH enzymes) in all hemipterans reflect the relevance of these effector molecules in insect defense. A number of catalases, GPx and TPx reveal the importance of antioxidant mechanisms in hemipterans.

Several AMPs were found in most species; although differences in AMP repertories were detected. Lysozymes related to digestive and immune defense functions were identified in all hemipterans. Defensins were detected only in the hematophagous hemipterans, exhibiting divergence according their differential expression in insect tissues. In contrast, no cecropins and attacins were detected in hemipterans, corroborating previous observations. Similarly, homologs of other *Drosophila* AMPs were not detected in any hemipteran species.

Most of the canonical components of the Toll and Jak-STAT signaling cascades are conserved in the studied insects. In contrast, key components of the Imd pathway (Imd, FADD and DREED) were absent from all hemipterans. Orthologs of Imd, FADD and DREED were documented in a number of holometabolous insects that undergo complete larval midgut remodeling of during metamorphosis. Conversely, the lack of Imd, FADD and DREED appears to be a common feature in more ancient insects with incomplete metamorphosis (hemimetabolous insects), including hemipterans. We speculate that these Imd signaling members were recruited by holometabolous insects in response to pathogenic bacterial threats during midgut remodeling.

## Additional file

Additional file 1: Table S1. Immunity-genes reference dataset*.* Dataset of immunity-genes sequences retrieved from GenBank, VectorBase, UniProt, FlyBase and Ensembl databases and published information used to query for immune-related homologs in TTTs transcriptomes and RPRO, CLEC and ACPI predicted-peptide sets. **Table S2.** Immune-related homologs involved in immune recognition and activation (PGRPs; GNBPs; Lectins; TEPs; Scavenger receptors; Clip-domain serine-proteases) and their respective transcriptome/genome sequence-IDs in the six hemipteran species. **Table S3.** Immune-related homologs involved in immune signaling (Toll, Jak-STAT and Imd pathways) and their respective transcriptome/genome sequence-IDs in the six hemipteran species. **Table S4.** Immune-related homologs involved in immune effectors (antimicrobial peptides; PPOs; nitric oxide synthase; NADPH enzymes) and their respective transcriptome/genome sequence-IDs in the six hemipteran species. **Table S5.** Immune-related homologs involved in immune regulation (immune negative regulators) and their respective transcriptome/genome sequence-IDs in the six hemipteran species. **Table S6.** Immune-related homologs involved in antioxidant mechanisms (Catalases; Peroxidases) and their respective transcriptome/genome sequence-IDs in the six hemipteran species. **Table S7.** Immune-related homologs involved in RNA interference (RNAi machinery components) and their respective transcriptome/genome sequence-IDs in the six hemipteran species. **Table S8.** Immune-related homologs involved in coagulation (Fondue and Hemolectin proteins) and their respective transcriptome/genome sequence-IDs in the six hemipteran species. (XLSX 53 kb)

## Abbreviations

ACPI: *Acyrthosiphon pisum*
Ago: argonaut protein
AMP: antimicrobial peptide
CLIP: CLIP domain serine proteases
CTL: C-type lectin
CysGPx: cysteine-containing glutathione peroxidase
Dcr: dicer protein
dsRNA: double-stranded RNA
Duox: dual oxidase
GNBP: Gram-negative binding protein
GPx: glutathione peroxidase
Imd: immunodeficiency
Jak-STAT: Janus kinase/Signal transducers and activators of transcription
Loqs: loquacious protein
NO: nitric oxide
Nox: NADPH oxidase
PAMP: pathogen-associated molecular pattern
PGRP: peptidoglycan receptor protein
PLCβ: phospholipase C-β
PNG: peptidoglycan
PO: phenoloxidase
PPO: prophenoloxidase
PRR: pathogen recognition receptor
Px: peroxidase
RLC: RISC-loading complex
RNAi: RNA interference
ROS: reactive oxygen species
RPRO: *Rhodnius prolixus*
siRNA: small interfering RNA
SR: scavenger receptor
SR-B: scavenger receptor class B
SR-C: scavenger receptor class C
TBRA: *Triatoma brasiliensis*
TDIM: *Triatoma dimidiata*
TEP: thioester-containing protein
TINF: *Triatoma infestans*
TPAL: *Triatoma pallidipennis*
TPx: thioredoxin peroxidase
TTTs: *Triatoma pallidipennis*, *Triatoma dimidiata* and *Triatoma infestans*
